# Differences in Pain Intensity of Tumors Spread to the Anterior versus Anterolateral/Lateral Portions of the Vertebral Body Based on CT Scans

**DOI:** 10.1155/2019/9387941

**Published:** 2019-05-13

**Authors:** Hui-Ching Hsu, Tzu-Yao Liao, Long-Sun Ro, Yu-Hsiang Juan, Chuang-Chi Liaw

**Affiliations:** ^1^Chinese Acupuncture and Traumatology, Department of Traditional Chinese Medicine, Chang-Gung Memorial Hospital and Chang-Gung University College, Taoyuan, Taiwan; ^2^Division of Hemato-Oncology, Department of Internal Medicine, Chang-Gung Memorial Hospital and Chang-Gung University College, Taoyuan, Taiwan; ^3^Department of Neurology, Chang-Gung Memorial Hospital and Chang-Gung University College, Taoyuan, Taiwan; ^4^Department of Medical Imaging and Intervention, Chang-Gung Memorial Hospital and Chang-Gung University College, Taoyuan, Taiwan

## Abstract

We investigated whether the intensity of cancer pain differs for malignant tumors that have spread to anterior or anterolateral/lateral portions of the vertebral body. We hypothesize that tumor spread to the anterolateral/lateral vertebral body elicits more serious pain due to increased irritation of the spinal nerve. The selection criteria were as follows: (1) advanced or metastatic solid tumor; (2) radicular pain without extremity weakness; (3) malignant lesions anteriorly, anterolaterally, or laterally located at the vertebral body either spread locoregionally or over a greater distance via metastasis based on CT scan diagnosis; and (4) patient needs to use opioids for pain relief. Severe spinal pain intensity was defined as spinal pain for which patients required either strong opioids or spinal irradiation for relief. Eighty-six patients were enrolled in the study. Bone lesions were mainly osteolytic. Thirty-nine tumors spread to the vertebral body in the anterior direction, and 47 in the anterolateral/lateral direction. Severe pain intensity related to vertebral body lesions was due to anterolateral/lateral spread, primary sites of nonurothelial carcinoma, metastatic vertebral lesions, multiple lesions within a vertebrum, and location within the cervical-thoracic spine. In conclusion, patients with tumor spread to the anterolateral/lateral portion of vertebrae bodies based on CT scan diagnosis experienced severe cancer pain. These patients needed strong opioids or palliative spinal irradiation for pain relief.

## 1. Introduction

Malignant tumors located laterally or anterolaterally within the vertebral body can cause pain because they involve the ventral ramus, which is the anterior division of a spinal nerve located at the anterolateral aspect of the spinal cord. It carries sensory and motor fibers to the anterolateral parts of the trunk and limbs. The ventral rami exit the anterolateral vertebral body and converge to form the nerve plexuses (cervical, brachial, and lumbosacral). Intercostal nerves also arise from the ventral rami of the thoracic spinal nerves. Radiculopathy is due to compressed or irritated nerve roots, which may cause pain, numbness, tingling, or weakness along the course of the nerve [1, 2]. Radicular pain is characterized by pain radiating along the dermatome of a nerve [3]. Back pain and radicular pain are the most common initial presentations of a malignant spinal cord compression [4]. Tumor spread to the anterolateral/lateral portions of the vertebral body may irritate the spinal nerve roots and ventral rami more so than tumors that spreads to the anterior vertebral body.

In this retrospective study, we investigated whether the intensity of cancer pain is different for malignant tumors that spread to anterior or anterolateral/lateral portions of the vertebral body based on CT scan diagnosis. We hypothesized that anterolateral/lateral tumors elicit more severe pain due to enhanced irritation of the nearby spinal nerve.

## 2. Materials and Methods

### 2.1. Study Population and Diagnostic Criteria

We conducted a retrospective case series study using data collected from patients from the oncology wards of Chang-Gung Memorial Hospital, Taoyuan, Taiwan, between January 2010 and February 2018. A single medical oncologist specialized in urological cancer provided most of the data, and the majority of the patients had urothelial carcinomas. The patients were all hospitalized as they needed to receive systemic therapy, radiotherapy, or the management of other complications. We conducted a retrospective chart review and performed computed tomography (CT) scans for patient tumor extent and their pain management.

The selection criteria included the following: (1) advanced or metastatic solid tumor; (2) radicular pain without extremity weakness; (3) malignant lesions anteriorly, anterolaterally, or laterally located at the vertebral body and either locoregionally spread or metastasized based on diagnosis using abdominal or chest CT scans; and (4) patients need to use opioids for pain relief. Radicular pain was defined as pain radiating along the dermatome of a nerve.

### 2.2. Pain Management

A visual analogue scale (VAS) on a 10 cm was used to measure cancer pain intensity. The scale numbered from zero to 10, zero corresponds to no pain and 10 to maximum pain felt by patients [5, 6]. The cutting point of cancer pain was mild (VAS = 1–3), moderate (VAS = 4–6), and severe (VAS = 7–10) [5, 6].

Analgesics were given according to the World Health Organization (WHO) ladder for cancer pain relief, with opioids and/or nonsteroidal anti-inflammatory drugs (NSAIDs, mainly naproxen) and/or adjuvant analgesia (mainly amitriptyline) [7]. Ultracet (combination of acetaminophen 325 mg and tramadol hydrochloride 37.5 mg) was used in patients with moderate cancer pain. We used strong opioids including morphine, hydromorphone, fentanyl, or oxycodone for relief in patients with severe cancer pain. We selected NSAIDs for mild cancer pain or additive NSAIDs when combined with opioids for moderate to severe cancer pain. We selected Ultracet for moderate cancer pain or failure to pain control from NSAIDs or acetaminophen. We selected strong opioids for severe cancer pain or failure to pain control from Ultracet. We selected adjuvant analgesics used for neuropathic pain which incompletely relieved by opioid analgesics.

Systemic therapy included chemotherapy, hormone therapy, or targeted therapy. Patients received palliative spinal irradiation for back pain and radicular pain in selected cases. Severe spinal pain intensity was defined as requiring either strong opioids or spinal irradiation to relieve pain. Moderate spinal pain intensity was defined as requiring Ultracet and not needing spinal irradiation to relieve pain.

The parameters for evaluating the severity of spinal cancer pain intensity included tumor spread to the vertebral body (anterolateral/lateral versus anterior), age (<65 years versus ≥ 65 years), sex, spinal location (cervical-thoracic versus lumbar-sacral), extent of disease spread to the vertebral body (locoregional versus distant metastases), number of tumors within one vertebral body (single versus multiple), and primary site of urothelial carcinoma (yes versus no).

### 2.3. Statistical Analysis

Continuous data (presented as mean ± standard deviation) were used for the VAS for pain severity analysis, including tumor spread to the vertebral body anterolaterally/laterally and anteriorly. We used the chi-squared test to detect differences between subgroups, and *p* < 0.05 was considered statistically significant.

## 3. Results

### 3.1. Patient Characteristics

The patients' clinical characteristics are shown in Table 1. Eighty-six patients with advanced or metastatic solid tumors were enrolled. The population consisted of 58 men and 28 women who ranged in age from 25 to 89 years (median, 65 years). Forty-nine (57%) patients had primary malignancies of urothelial cancer, including the renal pelvis (*n*=30), bladder (*n*=16), and ureters (*n*=3). The remaining 37 patients had the following cancer site: the lung (*n*=10), breast (*n*=4), prostate (*n*=5), renal (*n*=5), gastrointestinal (*n*=8), head and neck (*n*=2), and testicular (*n*=3).

### 3.2. Pain Patterns, Pain Intensity, and CT Scan Features

**Figure 1:**
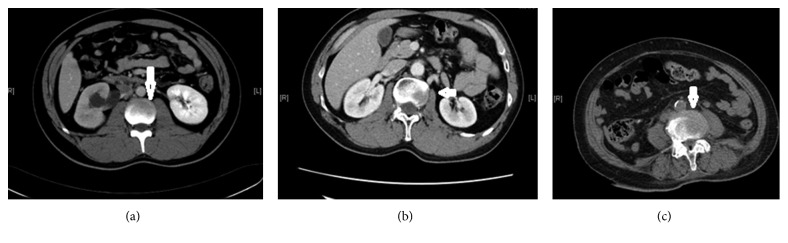
Tumor spread to the vertebral body. CT scans showing tumor spread to (a) the anterior portion, (b) the lateral portion, and (c) the anterolateral portion. Arrows indicate lesions and spreading routes within the vertebral body.

**Figure 2:**
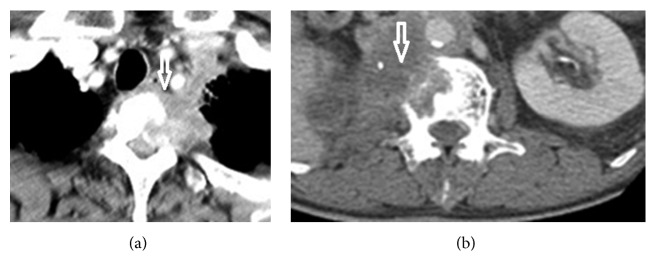
Primary tumor spread directly to the vertebral body. CT scans showing (a) lung squamous cell carcinoma and (b) large renal pelvis urothelial carcinoma that spread directly to the anterolateral portion of the vertebra. Arrows indicate lesions and spreading routes within the vertebral body.

**Figure 3:**
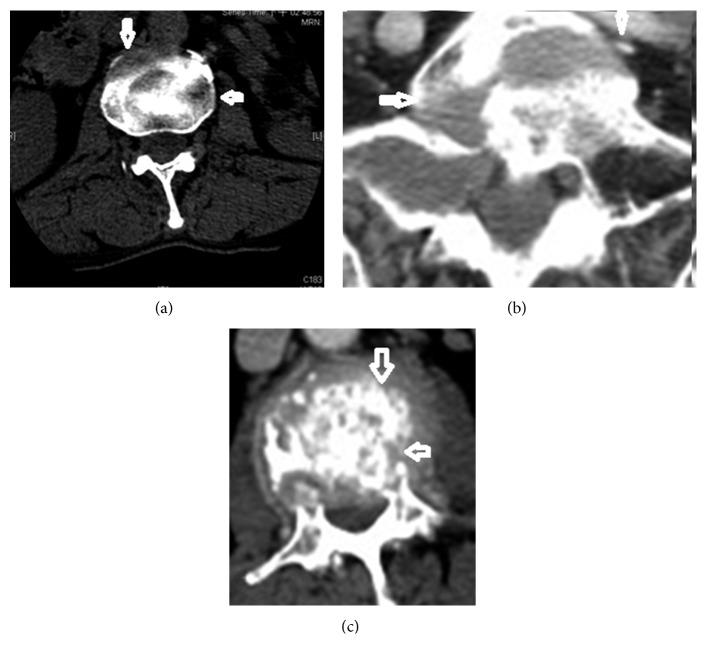
Tumor spread to the anterior and lateral portions of the vertebral body. (a–c) CT scans showing multiple lesions over the vertebral body. Arrows indicate lesions and spreading routes within the vertebral body.

**Figure 4:**
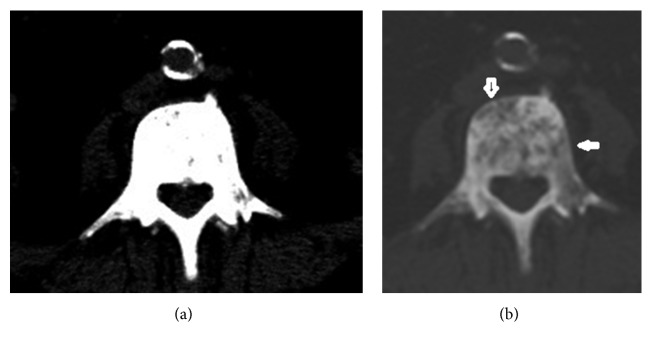
A case of prostate cancer with osteoblastic metastasis. (a, b) CT scan showing a bone window tumor with osteoblastic metastasis spread to anterior and lateral portions of the vertebra. Arrows indicate lesions and spreading routes within the vertebral body.

The mean VAS for pain intensity was 8.3 ± 1.3 (range, 4 to 10) for tumor spread to the vertebral body anterolaterally/laterally. The mean VAS for pain intensity was 4.8 ± 0.8 (range, 4 to 7) for tumor spread to the vertebral body anteriorly. Vertebral body lesions due to anterior spread (Figure 1(a)), lateral spread (Figure 1(b)), or anterolateral spread (Figure 1(c)) were demonstrated in 39 (45%), 22 (26%), and 25 (29%) patients, respectively. Disease with locoregional spread (Figure 2) was noted in 50 (58%) patients. Multiple (≥2) lesions at the vertebral body were detected in 22 (26%) patients (Figure 3). Lumbar-sacral locations were noted in 72 (84%) patients. Eighty-two (95%) patients had osteolytic bone lesions, while two had osteoblastic, and one had mixed osteolytic-osteoblastic bone patterns (Figure 4).

For back and radicular cancer pain relief, 43 patients used Ultracet alone, 20 used strong opioids, 13 of 43 patients used Ultracet groups adding palliative spinal irradiation, and 10 of 20 patients used strong opioids receiving palliative spinal irradiation. We then divided patients into those with severe pain intensity and those with moderate pain intensity based on the pain management they received, as mentioned above. Forty-three patients were grouped as experiencing severe pain intensity, and the other 43 were grouped as moderate pain intensity.

### 3.3. Factors Associated with Patient Need for the Use of Strong Opioids or Palliative Spinal Irradiation for Pain Management

The assessment of parameters related to pain intensity among patients with tumor spread to the vertebral body is shown in Table 2. We found that pain intensity related to vertebral body lesions was significantly affected by anterior versus lateral or anterolateral spreading (*p* < 0.00001), urothelial versus nonurothelial carcinoma (*p* < 0.00001), direct locoregional spread versus distant metastases (*p* < 0.00001), lesion number (single versus ≥ 2, *p*=0.0004), and lumbar-sacral versus cervical-thoracic spinal location (*p*=0.0004).

Thirty-nine patients were diagnosed with tumor spread to the anterior vertebral body. Of these patients, 37 (95%) had moderate pain intensity, 38 (97%) had tumors in the lumbar-sacral area, 28 (72%) had a primary site of urothelial carcinoma, 32 (82%) had tumors that were locoregionally spread, and 37 (95%) had single vertebral body lesions. Thirty-five (90%) patients received systemic therapy, and none among them received palliative spinal irradiation.

Forty-seven patients were diagnosed with tumor spread to the anterolateral/lateral portions of the vertebral body and 41 (87%) had severe pain intensity. These patients included 13 of 14 (93%) with cervical-thoracic spine location, 31 of 37 (84%) with primary site of nonurothelial carcinoma, 30 of 37 (81%) with metastatic vertebral body lesion, and 20 of 22 (91%) with ≥2 lesions within one vertebral body. Two patients had osteoblastic and one mixed osteolytic-osteoblastic bone patterns. Twenty-three (49%) of these severe pain patients received palliative spinal irradiation and 41 (83%) received systemic therapy.

## 4. Discussion

Radicular pain is a type of pain radiating along the dermatome of a nerve. Radicular pain is caused by compression, inflammation, and/or injury to a spinal nerve root. Management of cancer pain is multimodal, as pain is usually a combination of inflammation and irritation. Besides opioids, physicians often prescribe NSAIDs and/or adjuvant analgesia for neuropathic pain relief [8–12].

All were metastatic or advanced tumors. But cancer pain depends on tumor location. For pain intensity, we must emphasize that tumor location was more important than those with advanced tumor or disseminated metastases. Tumors that spread to the anterolateral/lateral portion of the vertebral body irritate spinal nerve roots more than those that spread to the anterior portion. In this study, we detected a significant difference in pain intensity between anterolateral/lateral and lateral tumor spread based on CT scans. The presence of multiple lesions within one vertebral body leads to enhanced irritation of the spinal nerve root compared to a single lesion.

Pain in cancer may arise from spinal nerve root by a tumor compressing or infiltrating nearby the lesion, related to their degree and area of osteolytic site. These thoughts are important but difficult in the assessment from CT scan.

Most of the patients with tumor spread to the anterior portion of the vertebral body exhibited moderate pain intensity, a lumbar-sacral location, primary site of urothelial carcinoma and locoregional spreading. Urothelial carcinoma typically spreads locoregionally and invades the vertebral body, a process that is probably linked to the development of bone metastases [13]. Pain intensity at location of these anterior spread tumors was mainly lumbar-sacral, and pain was usually moderate. Pain control with Ultracet and systemic therapy was adequate, and there was no need for palliative spinal irradiation in most cases. Tramadol centrally acts on opioid receptors and inhibits the action of norepinephrine and serotonin at the synapse for the management of cancer pain [14]. Ultracet, a tramadol/acetaminophen fixed-dose combination was reported to efficacious and safety in the therapy of breakthrough cancer pain [15]. However, tramadol with or without paracetamol (acetaminophen) for cancer pain is evidenced from randomized controlled trials that tramadol-produced pain relief is not as effective as morphine [16].

Conversely, most patients with anterolateral/lateral tumors that spread to the vertebral body experienced severe pain. Pain control required strong opioids or palliative spinal irradiation, in addition to systemic therapy in most cases.

Other management can improve spinal cancer pain. These include effective systemic therapies such as chemotherapy, targeted therapy, or hormone therapy to let tumor response and palliative spinal surgery. Vertebroplasty and kyphoplasty are conservative treatments with high rates of spinal cancer pain relief and vertebral body stabilization.

There are some limitations to the current study. First, this study is limited by its retrospective nature. Second, all subjects were in our practice and at a single referral medical center. Third, the majority of cancers were urological, as we specialize in urological cancer.

## 5. Conclusions

Patients with tumors that spread to the anterolateral/lateral portion of the vertebral body experienced more severe pain intensity than those with tumors that spread to the anterior portion based on CT scan diagnosis. These patients required strong opioids or palliative spinal irradiation for pain relief.

## Figures and Tables

**Table 1:** Clinical characteristics of 86 patients with tumor spread to the vertebral body.

Characteristics	No. of patients (%)
Age (years)	
Median (range)	65 (25–89)
Sex	
Male/female	58/28
Primary site of tumor	
Urothelial carcinoma	49 (57)
Nonurothelial carcinoma	37 (43)
Disease extent to the vertebral body	
Locoregional spread	50 (58)
Distant metastases	36 (42)
Tumor spread to the vertebral body	
Anterior	39 (45)
Lateral	22 (26)
Anterior and lateral	25 (29)
Spine location	
Cervical-thoracic	14 (16)
Lumbar-sacral	72 (84)
Number tumor involvement of one vertebral body	
Single	64 (74)
Multiple (≥2)	22 (26)
Pattern of bone metastasis	
Osteolytic	82 (95)
Osteoblastic	3 (3)
Mixed osteolytic with osteoblastic	1 (1)
Pain intensity	
Moderate^*∗*^	
Severe^	

^*∗*^need Ultracet for pain relief. ^need strong opioids or palliative spinal irradiation for pain relief.

**Table 2:** Parameters related to severe pain intensity among 86 patients with tumor spread to the vertebral body.

Characteristics	No./total no. (%)	*p* value
Age		0.66
<65	20/38 (53)	
≥65	23/48 (48)	
Sex		0.35
Male	16/28 (57)	
Female	27/58 (47)	
Site of spinal location		0.0004
Cervical-thoracic	13/14 (93)	
Lumbar-sacral	30/72 (42)	
Number of tumors of one vertebral body		0.00009
Single	23/64 (36)	
Multiple	20/22 (91)	
Tumor spread to vertebral body		<0.00001
Anterior	2/39 (5)	
Lateral	17/22 (77)	
Anterior and lateral	24/25 (96)	
Disease extent to the vertebral body		<0.00001
Locoregional spread	13/50 (26)	
Distant metastases	30/36 (83)	
Urothelial carcinoma		<0.00001
Yes	11/49 (22)	
No	32/37 (86)	

## Data Availability

The data used to support the findings of this study are available from the corresponding author upon request.
